# 4-Methyl-2-oxo-2,3,4,5-tetra­hydro-1*H*-1,5-benzodiazepine-5-carbaldehyde

**DOI:** 10.1107/S1600536809034825

**Published:** 2009-09-05

**Authors:** K. Ravichandran, P. Sakthivel, S. Ponnuswamy, P. Ramesh, M. N. Ponnuswamy

**Affiliations:** a Centre of Advanced Study in Crystallography and Biophysics, University of Madras, Guindy Campus, Chennai 600 025, India; b Department of Chemistry, Government Arts College (Autonomous), Coimbatore 641 018, India

## Abstract

In the title compound, C_11_H_12_N_2_O_2_, a benzodiazepine derivative, the seven-membered ring adopts a distorted boat conformation. The crystal packing is controlled by inter­molecular N—H⋯O and C—H⋯O inter­actions.

## Related literature

For the hypnotic effects of benzodiazepines, see: Gringauz (1999 ▶). For their use in the treatment of gastrointestinal and central nervous system disorders, see: Rahbaek *et al.* (1999 ▶). For other therapeutic applications, see: Albright *et al.* (1998 ▶); Lee *et al.* (1999 ▶). For hydrogen-bond motifs, see: Bernstein *et al.* (1995 ▶). For puckering and asymmetry parameters, see: Cremer & Pople (1975 ▶);) ; Nardelli (1983 ▶). For details of the preparation, see: Venkatraj *et al.* (2008 ▶).
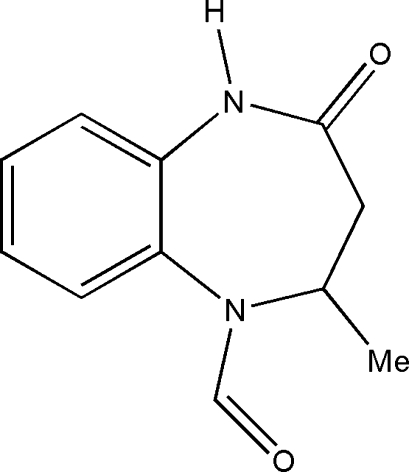


## Experimental

### 

#### Crystal data

C_11_H_12_N_2_O_2_
*M* *_r_* = 204.23
Monoclinic, 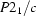

*a* = 5.3284 (1) Å
*b* = 12.9387 (4) Å
*c* = 14.6227 (5) Å
β = 97.968 (2)°
*V* = 998.39 (5) Å^3^
*Z* = 4
Mo *K*α radiation
μ = 0.10 mm^−1^
*T* = 293 K
0.30 × 0.20 × 0.15 mm

#### Data collection

Bruker Kappa APEXII area-detector diffractometer
Absorption correction: multi-scan (*SADABS*; Sheldrick, 2001 ▶) *T*_min_ = 0.977, *T*_max_ = 0.986
14697 measured reflections
3727 independent reflections
2453 reflections with *I* > 2σ(*I*)
*R*_int_ = 0.028

#### Refinement

*R*[*F* ^2^ > 2σ(*F* ^2^)] = 0.055
*wR*(*F* ^2^) = 0.163
*S* = 1.05
3727 reflections
141 parameters
H atoms treated by a mixture of independent and constrained refinement
Δρ_max_ = 0.40 e Å^−3^
Δρ_min_ = −0.17 e Å^−3^

### 

Data collection: *APEX2* (Bruker, 2004 ▶); cell refinement: *SAINT* (Bruker, 2004 ▶); data reduction: *SAINT*; program(s) used to solve structure: *SHELXS97* (Sheldrick, 2008 ▶); program(s) used to refine structure: *SHELXL97* (Sheldrick, 2008 ▶); molecular graphics: *ORTEP-3* (Farrugia, 1997 ▶); software used to prepare material for publication: *SHELXL97* and *PLATON* (Spek, 2009 ▶).

## Supplementary Material

Crystal structure: contains datablocks global, I. DOI: 10.1107/S1600536809034825/bt5041sup1.cif

Structure factors: contains datablocks I. DOI: 10.1107/S1600536809034825/bt5041Isup2.hkl

Additional supplementary materials:  crystallographic information; 3D view; checkCIF report

## Figures and Tables

**Table 1:** Hydrogen-bond geometry (Å, °)

*D*—H⋯*A*	*D*—H	H⋯*A*	*D*⋯*A*	*D*—H⋯*A*
N1—H1⋯O2^i^	0.89 (2)	2.12 (2)	2.9745 (16)	161.3 (18)
C13—H13⋯O1^ii^	0.93	2.38	3.2220 (18)	150

Symmetry codes: (i) 
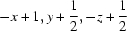
; (ii) 
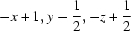
.

**Fig. 1.**
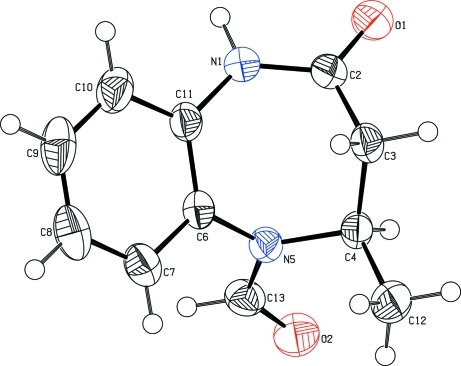
Perspective view of the molecule showing the thermal ellipsoids are drawn at 50% probability level.

**Fig. 2.**
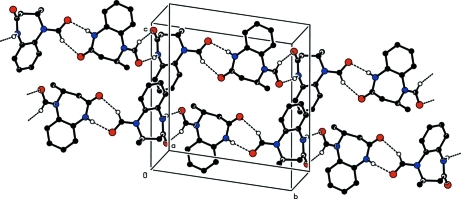
The crystal packing of the molecules viewed down a–axis. H atoms not involved in hydrogen bonding have been omitted for clarity.

## supplementary crystallographic information

### Comment

Benzodiazepines are used for the purpose of hypnotic effects, owing to their less toxic and less severe withdrawal effects when compared with barbiturates (Gringauz, 1999). Benzodiazepines from *aspergillus* include *asperlicin*, which is used for treatment of gastrointestinal and central nervous system (*CNS*) disorders (Rahbaek *et al.*,1999). The other therapeutic applications (Lee *et al.*, 1999) of benzodiazepines include vasopressin antagonists (Albright *et al.*, 1998). In view of these importance and to ascertain the molecular conformation, crystallographic study of the title compound has been carried out.

The *ORTEP* diagram of the title compound is shown in Fig.1. The benzodiazepine ring adopts a distorted boat conformation. The puckering parameters (Cremer & Pople, 1975) and the asymmetry parameters (Nardelli, 1983) for this ring are q_2_ = 0.933 (1) Å, q_3_ = 0.170 (1) Å, φ_2_ = 144.8 (1)°, φ_3_ = 18.0 (4)° and Δ2(C4)=13.2 (1)°. The sum of the bond angles at N1(359.8°) and N5(359.8°) of the benzodiazepine ring is in accordance with *sp*^2^ hybridization.

The crystal packing is controlled by N—H···O and C—H···O types of intermolecular interactions in addition to van der Waals forces. Atom N1 at (*x*, *y*, *z*) donates a proton to O2 (1 - *x*, 1/2 + *y*, 1/2 - *z*), which forms a C7 (Bernstein *et al.*, 1995) one dimensional chain running along b–axis. The intermolecular hydrogen bond C13—H13···O1 also connects the molecule into another C7 chain running along b–axis. Thus the combination of N—H···O and C—H···O intermolecular hydrogen bonds form a graph set motif of *R*_2_^2^(7) dimer to stabilize the molecules and extend along b–axis (Fig. 2).

### Experimental

An ice-cold solution of acetic-formic anhydride was prepared from acetic anhydride (10 ml) and 85% formic acid (5 ml) was added slowly to a cold solution of tetrahydrobenzodiazepin-2-one (0.88 g) in anhydrous benzene (30 ml). The reaction mixture was stirred at room temperature for 4 hrs. The organic layer was separated, dried over anhydrous Na_2_SO_4_ and concentrated. The resulting mass was purified by crystallization from benzene-petroleum ether (333–353° K) mixture (1:1) (Venkatraj *et al.*, 2008)

### Refinement

The   H atom bonded to N was freely refined whereas the other H atoms were positioned geometrically (C—H=0.93–0.98 Å) and allowed to ride on their parent atoms, with 1.5*U*_eq_(C) for methyl H and 1.2 *U*_eq_(C) for other H atoms.

### Crystal data

**Table:**

C_11_H_12_N_2_O_2_	*F*(000) = 432
*M**_r_* = 204.23	*D*_x_ = 1.359 Mg m^−^^3^
Monoclinic, *P*2_1_/*c*	Mo *K*α radiation, λ = 0.71073 Å
Hall symbol: -P 2ybc	Cell parameters from 2563 reflections
*a* = 5.3284 (1) Å	θ = 2.1–32.9°
*b* = 12.9387 (4) Å	µ = 0.10 mm^−^^1^
*c* = 14.6227 (5) Å	*T* = 293 K
β = 97.968 (2)°	Block, colourless
*V* = 998.39 (5) Å^3^	0.30 × 0.20 × 0.15 mm
*Z* = 4	

### Data collection

**Table:**

Bruker Kappa APEXII area-detector diffractometer	3727 independent reflections
Radiation source: fine-focus sealed tube	2453 reflections with *I* > 2σ(*I*)
graphite	*R*_int_ = 0.028
ω and φ scans	θ_max_ = 32.9°, θ_min_ = 2.1°
Absorption correction: multi-scan (*SADABS*; Sheldrick, 2001)	*h* = −8→8
*T*_min_ = 0.977, *T*_max_ = 0.986	*k* = −19→19
14697 measured reflections	*l* = −22→12

### Refinement

**Table:**

Refinement on *F*^2^	Primary atom site location: structure-invariant direct methods
Least-squares matrix: full	Secondary atom site location: difference Fourier map
*R*[*F*^2^ > 2σ(*F*^2^)] = 0.055	Hydrogen site location: inferred from neighbouring sites
*wR*(*F*^2^) = 0.163	H atoms treated by a mixture of independent and constrained refinement
*S* = 1.05	*w* = 1/[σ^2^(*F*_o_^2^) + (0.076*P*)^2^ + 0.1596*P*] where *P* = (*F*_o_^2^ + 2*F*_c_^2^)/3
3727 reflections	(Δ/σ)_max_ = 0.004
141 parameters	Δρ_max_ = 0.40 e Å^−^^3^
0 restraints	Δρ_min_ = −0.17 e Å^−^^3^

### Special details

**Table:**

Geometry. All e.s.d.'s (except the e.s.d. in the dihedral angle between two l.s. planes) are estimated using the full covariance matrix. The cell e.s.d.'s are taken into account individually in the estimation of e.s.d.'s in distances, angles and torsion angles; correlations between e.s.d.'s in cell parameters are only used when they are defined by crystal symmetry. An approximate (isotropic) treatment of cell e.s.d.'s is used for estimating e.s.d.'s involving l.s. planes.
Refinement. Refinement of *F*^2^ against ALL reflections. The weighted *R*-factor *wR* and goodness of fit *S* are based on *F*^2^, conventional *R*-factors *R* are based on *F*, with *F* set to zero for negative *F*^2^. The threshold expression of *F*^2^ > σ(*F*^2^) is used only for calculating *R*-factors(gt) *etc*. and is not relevant to the choice of reflections for refinement. *R*-factors based on *F*^2^ are statistically about twice as large as those based on *F*, and *R*- factors based on ALL data will be even larger.

### Fractional atomic coordinates and isotropic or equivalent isotropic displacement parameters (Å^2^)

**Table:**

	*x*	*y*	*z*	*U*_iso_*/*U*_eq_	
O1	0.5676 (2)	0.55730 (10)	0.36634 (8)	0.0596 (3)	
O2	0.4734 (2)	0.15716 (8)	0.36319 (8)	0.0498 (3)	
N1	0.3101 (2)	0.49320 (9)	0.24515 (8)	0.0411 (3)	
H1	0.395 (4)	0.5303 (15)	0.2083 (14)	0.060 (5)*	
C2	0.3797 (3)	0.50642 (10)	0.33737 (10)	0.0391 (3)	
C3	0.2127 (3)	0.45788 (10)	0.39982 (9)	0.0370 (3)	
H3A	0.0372	0.4725	0.3762	0.044*	
H3B	0.2490	0.4889	0.4606	0.044*	
C4	0.2483 (2)	0.34179 (10)	0.40847 (8)	0.0320 (3)	
H4	0.4214	0.3286	0.4385	0.038*	
N5	0.22002 (19)	0.29427 (8)	0.31600 (7)	0.0319 (2)	
C6	0.0534 (2)	0.33715 (10)	0.24086 (8)	0.0313 (3)	
C7	−0.1530 (3)	0.27966 (12)	0.19987 (10)	0.0421 (3)	
H7	−0.1912	0.2168	0.2256	0.051*	
C8	−0.3011 (3)	0.31534 (14)	0.12141 (11)	0.0522 (4)	
H8	−0.4377	0.2764	0.0938	0.063*	
C9	−0.2459 (3)	0.40821 (15)	0.08435 (11)	0.0552 (4)	
H9	−0.3435	0.4315	0.0306	0.066*	
C10	−0.0470 (3)	0.46775 (12)	0.12580 (10)	0.0479 (4)	
H10	−0.0138	0.5315	0.1006	0.057*	
C11	0.1037 (2)	0.43294 (10)	0.20486 (9)	0.0343 (3)	
C12	0.0669 (3)	0.29350 (14)	0.46723 (11)	0.0503 (4)	
H12A	−0.1043	0.3047	0.4387	0.075*	
H12B	0.0909	0.3245	0.5274	0.075*	
H12C	0.0990	0.2206	0.4728	0.075*	
C13	0.3356 (3)	0.20421 (10)	0.30322 (10)	0.0388 (3)	
H13	0.3090	0.1754	0.2444	0.047*	

### Atomic displacement parameters (Å^2^)

**Table:**

	*U*^11^	*U*^22^	*U*^33^	*U*^12^	*U*^13^	*U*^23^
O1	0.0699 (7)	0.0577 (7)	0.0464 (7)	−0.0305 (6)	−0.0085 (5)	0.0062 (5)
O2	0.0557 (6)	0.0390 (5)	0.0529 (7)	0.0129 (4)	0.0013 (5)	0.0044 (5)
N1	0.0551 (7)	0.0357 (6)	0.0325 (6)	−0.0110 (5)	0.0055 (5)	0.0007 (5)
C2	0.0496 (7)	0.0299 (6)	0.0359 (7)	−0.0042 (5)	−0.0012 (5)	0.0001 (5)
C3	0.0445 (7)	0.0363 (6)	0.0298 (6)	0.0006 (5)	0.0036 (5)	−0.0063 (5)
C4	0.0333 (5)	0.0362 (6)	0.0259 (5)	−0.0012 (4)	0.0019 (4)	−0.0012 (5)
N5	0.0358 (5)	0.0295 (5)	0.0297 (5)	0.0016 (4)	0.0026 (4)	−0.0011 (4)
C6	0.0319 (5)	0.0347 (6)	0.0269 (6)	0.0025 (4)	0.0027 (4)	−0.0038 (5)
C7	0.0389 (7)	0.0466 (8)	0.0404 (7)	−0.0060 (5)	0.0034 (5)	−0.0092 (6)
C8	0.0389 (7)	0.0694 (10)	0.0453 (8)	0.0016 (7)	−0.0052 (6)	−0.0185 (8)
C9	0.0544 (9)	0.0720 (11)	0.0348 (7)	0.0229 (8)	−0.0100 (6)	−0.0063 (7)
C10	0.0634 (9)	0.0443 (8)	0.0337 (7)	0.0138 (7)	−0.0012 (6)	0.0021 (6)
C11	0.0407 (6)	0.0338 (6)	0.0278 (6)	0.0033 (5)	0.0027 (5)	−0.0041 (5)
C12	0.0563 (9)	0.0571 (9)	0.0397 (8)	−0.0147 (7)	0.0142 (6)	0.0001 (7)
C13	0.0428 (7)	0.0298 (6)	0.0436 (7)	0.0026 (5)	0.0055 (5)	−0.0019 (5)

### Geometric parameters (Å, °)

**Table:**

O1—C2	1.2240 (16)	C6—C11	1.3872 (18)
O2—C13	1.2248 (17)	C6—C7	1.3925 (17)
N1—C2	1.3589 (18)	C7—C8	1.379 (2)
N1—C11	1.4088 (17)	C7—H7	0.9300
N1—H1	0.89 (2)	C8—C9	1.367 (3)
C2—C3	1.498 (2)	C8—H8	0.9300
C3—C4	1.5170 (18)	C9—C10	1.381 (2)
C3—H3A	0.9700	C9—H9	0.9300
C3—H3B	0.9700	C10—C11	1.3881 (18)
C4—N5	1.4740 (16)	C10—H10	0.9300
C4—C12	1.5145 (19)	C12—H12A	0.9600
C4—H4	0.9800	C12—H12B	0.9600
N5—C13	1.3430 (16)	C12—H12C	0.9600
N5—C6	1.4259 (15)	C13—H13	0.9300
			
C2—N1—C11	125.06 (12)	C8—C7—C6	120.40 (14)
C2—N1—H1	116.2 (13)	C8—C7—H7	119.8
C11—N1—H1	118.5 (13)	C6—C7—H7	119.8
O1—C2—N1	120.58 (14)	C9—C8—C7	119.65 (14)
O1—C2—C3	122.74 (13)	C9—C8—H8	120.2
N1—C2—C3	116.67 (12)	C7—C8—H8	120.2
C2—C3—C4	112.83 (11)	C8—C9—C10	120.70 (14)
C2—C3—H3A	109.0	C8—C9—H9	119.6
C4—C3—H3A	109.0	C10—C9—H9	119.6
C2—C3—H3B	109.0	C9—C10—C11	120.32 (15)
C4—C3—H3B	109.0	C9—C10—H10	119.8
H3A—C3—H3B	107.8	C11—C10—H10	119.8
N5—C4—C12	110.87 (11)	C6—C11—C10	119.08 (13)
N5—C4—C3	109.94 (10)	C6—C11—N1	121.10 (11)
C12—C4—C3	111.93 (12)	C10—C11—N1	119.78 (13)
N5—C4—H4	108.0	C4—C12—H12A	109.5
C12—C4—H4	108.0	C4—C12—H12B	109.5
C3—C4—H4	108.0	H12A—C12—H12B	109.5
C13—N5—C6	118.62 (11)	C4—C12—H12C	109.5
C13—N5—C4	119.93 (11)	H12A—C12—H12C	109.5
C6—N5—C4	121.21 (10)	H12B—C12—H12C	109.5
C11—C6—C7	119.78 (12)	O2—C13—N5	124.93 (13)
C11—C6—N5	120.50 (10)	O2—C13—H13	117.5
C7—C6—N5	119.61 (12)	N5—C13—H13	117.5
			
C11—N1—C2—O1	177.36 (14)	N5—C6—C7—C8	−173.39 (13)
C11—N1—C2—C3	−3.9 (2)	C6—C7—C8—C9	−0.7 (2)
O1—C2—C3—C4	−105.49 (15)	C7—C8—C9—C10	−1.4 (2)
N1—C2—C3—C4	75.77 (15)	C8—C9—C10—C11	1.4 (2)
C2—C3—C4—N5	−52.89 (14)	C7—C6—C11—C10	−2.81 (19)
C2—C3—C4—C12	−176.57 (11)	N5—C6—C11—C10	173.34 (12)
C12—C4—N5—C13	−81.79 (15)	C7—C6—C11—N1	179.59 (12)
C3—C4—N5—C13	153.92 (12)	N5—C6—C11—N1	−4.25 (18)
C12—C4—N5—C6	92.50 (14)	C9—C10—C11—C6	0.7 (2)
C3—C4—N5—C6	−31.80 (15)	C9—C10—C11—N1	178.38 (13)
C13—N5—C6—C11	−118.66 (14)	C2—N1—C11—C6	−42.1 (2)
C4—N5—C6—C11	66.98 (16)	C2—N1—C11—C10	140.35 (15)
C13—N5—C6—C7	57.50 (17)	C6—N5—C13—O2	−176.50 (13)
C4—N5—C6—C7	−116.86 (13)	C4—N5—C13—O2	−2.1 (2)
C11—C6—C7—C8	2.8 (2)		

### Hydrogen-bond geometry (Å, °)

**Table:**

*D*—H···*A*	*D*—H	H···*A*	*D*···*A*	*D*—H···*A*
N1—H1···O2^i^	0.89 (2)	2.12 (2)	2.9745 (16)	161.3 (18)
C13—H13···O1^ii^	0.93	2.38	3.2220 (18)	150

Symmetry codes: (i) −*x*+1, *y*+1/2, −*z*+1/2; (ii) −*x*+1, *y*−1/2, −*z*+1/2.
